# Coexposure of Micro and Nano-Plastics with Pesticides: Cytotoxicity and Bioaccumulation Effects on a Fish Intestinal Cell Line

**DOI:** 10.1021/acs.est.5c14140

**Published:** 2025-12-29

**Authors:** Justin Scott, Estefanía Pereira Pinto, Kyle Forsythe, Kendra Hess, Jason Belden, Jorge Gonzalez-Estrella, Matteo Minghetti

**Affiliations:** † School of Civil and Environmental Engineering, 7618 Oklahoma State University, Stillwater, Oklahoma 74078, United States; ‡ Department of Biology, Oklahoma State University, Stillwater, Oklahoma 74078, United States; § Centro de Investigación Mariña, Departamento de Ecoloxía e Bioloxía Animal, Laboratorio de Ecoloxía Costeira (ECOCOST), 16784 Universidade de Vigo, Vigo 36310, Spain

**Keywords:** micro- and nanoplastics, rtgutgc, lysosomal integrity, sorption, ecotoxicology

## Abstract

Micro- and nanoplastics (MNPs) occur in aquatic environments and accumulate in fish. MNPs can also adsorb other contaminants present in aquatic environments, and there is limited information on exposure scenarios involving MNP and pesticide mixtures. Ultraviolet (UV) radiation and chemical oxidation of MNPs can affect the sorption properties of MNPs and chemicals, thus altering the exposure and effects on fish. Our study investigated the toxicity and bioaccumulation of a lindane and dichlorodiphenyldichloroethylene (DDE) mixture adsorbed onto pristine and weathered polyethylene (PE) MNPs. Three different PE MNP types were used: microplastics (2–10 μm), oxidized microplastics (10–15 μm), and a MNP mixture (0.2–9.9 μm), and additionally each type was UV-aged for comparisons. RTgutGC cells, derived from rainbow trout (*Oncorhynchus mykiss*) intestine, were used to evaluate the role of the particle type on pesticides bioaccumulation and toxicity. Results showed that UV aging did not affect the agglomeration in solution but decreased the MNP’s capacity to adsorb the pesticides (i.e., non-aged adsorbed 35% and 69% and UV-aged adsorbed 9.7% and 63% of lindane and DDE, respectively) likely due to a shift in MNPs hydrophobicity and consequently reduced the cytotoxicity of the pesticide MNPs mixture. Nanoplastics induced approximately 20% more lysosomal damage than microplastics, suggesting a distinct toxicity mechanism. Fluorescently labeled MNPs accumulated in intestinal cells which confirmed the internalization. Finally, bioaccumulation of DDE decreased approximately 2 to 8-fold in cells coexposed with all particle types, although lindane was not detected in the cells. Overall, our study indicated that MP and NPs reduce bioavailability of pesticides, but UV aging and particle fragmentation to nano size increased their bioaccumulation and toxicity in fish intestinal cells.

## Graphical abstract

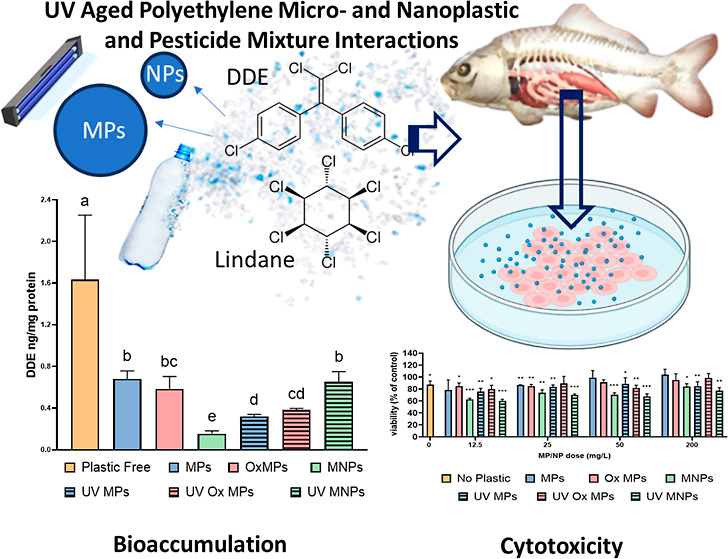

## 1 Introduction

Plastic pollution impacts aquatic ecosystems^1^ making it essential to understand plastic’s harmful effects on aquatic organisms amid the continuous rising global plastic output.^2,3^ Anthropogenic activities release plastics such as polyethylene (PE) into both marine^4,5^ and freshwater.^6–8^ In the environment, plastics break down into smaller fragments generating both microplastics (MPs < 5 mm) and nanoplastics (NPs < 1 μm).^9^ Micro- and nanoplastic (MNPs) continue to accumulate in aquatic environments and impact different trophic levels including mollusks, crustaceans, fish, sea birds, and dolphins.^10^

Fish are a primary example of organisms impacted by MNPs and studies have reported that exposure to MNPs can adversely affect fish populations.^11,12^ Ingestion of MNPs can affect invertebrate and vertebrate species^13^ and can result in trophic transfer across aquatic biota.^14^ Invertebrate to vertebrate trophic interactions such as algae, zooplankton, and fish^15–18^ ^,^ in aquatic systems may accumulate and ultimately affect human health.^19–22^ Moreover, MNPs coexist with legacy pollutants (e.g., metals and pesticides); however, there is a lack of information regarding both the MNP affinity to other pollutants and the MNP role on pollutant fate and toxicity in aquatic systems.^23^

While the body of literature on MNPs in aquatic environments continues to increase, due to MNPs complex physical and chemical properties, several questions remain to fully understand their environmental impact^10^ including (i) size effect of the particles (i.e., MPs vs NPs) and (ii) role of environmental weathering (i.e., weathering by chemical oxidation and UV radiation) on physical chemical properties of the particles and their environmental reactions. Moreover, research needs to further address environmentally relevant reactions including affinity of MNPs to priority pollutants (i.e., bioaccumulation of chemicals and toxicological insults in organisms).

Environmental weathering modifies the chemical and physical properties of the plastics. Particle fragmentation generates MNPs^24,25^ but nanoplastics are more difficult to study due to limitations in analytical methods.^26,27^ Weathering processes such as chemical oxidation and UV radiation can modify the functional chemistry of the MNPs that might affect their reactivity.^28,29^ Moreover, weathering processes affect the sorption of chemicals in the environment and increase the leaching of chemicals from MNPs.^30^ For instance, previous studies have shown that environmental weathering increased the adsorption of heavy metals such as cadmium (Cd) onto MNPs; however, the adsorption process decreased the bioavailability and toxicity of Cd fish intestinal cells.^31^ Due to their lipophilicity, pesticides can sorb onto MNPs in aquatic environments.^32,33^ However, in scenarios where MNPs and multiple pesticide mixtures co-occur, the sorption capacity of MNPs will likely change. Thus, the role of environmental weathering on the reactivity of MNPs and its influence on the uptake and toxicity of pesticides in mixed scenarios require further investigation.

MNPs may act as trojan horse of pesticides and increase the chemical uptake into aquatic organisms.^34^ A previous study has shown that MNPs can adsorb chemical toxicants^35^ and thereby increase exposure and uptake into the organism via ingestion.^36^ Studies effectively used the cell line RTgutGC, derived from the rainbow trout (Oncorhynchus mykiss) intestine, to evaluate the impact of metals,^37,38^ organic chemicals,^39,40^ and nanometals.^41,42^ Intestinal cells are relevant for MNP toxicity investigations as ingestion is a major route of exposure for fish.^43^ However, the complexity of aqueous media, materials used for experimentation in vitro, and the affinity of organic compounds toward plastic materials pose a challenge to the experimental design. One concern is that most experiments for aquatic exposures are conducted using plastic materials (e.g., tips and exposure vessels), which might adsorb the chemicals being studied. Current in vitro methods using cell lines for cytotoxicity assays typically use multiwell plates manufactured out of plastic materials (e.g., polyethylene, polystyrene, or polyvinyl chloride).^44,45^ Examples include the use of polyethylene materials for measuring bioaccumulation of metals^37^ and cytotoxicity of polar organics.^46,47^ However, toxicity and bioaccumulation of nonpolar organics can be difficult to measure due to partitioning of the chemicals into the plastic wells due to their hydrophobicity/lipophilicity and octanol–water partition coefficients.^48,49^ Therefore, research evaluating the sorption and toxicity of mixtures of organic compounds and MNPs must consider the affinity of the chemical to the vessel that is being used to carry out the experiment. Thus, in this study, we evaluated a novel procedure using only glass to reduce partitioning out of the exposure system and allowing the interaction of hydrophobic chemicals with the MNPs in solution and with cells.

Our study aimed to (i) assess the adsorption of a dichlorodiphenyldichloroethylene (DDE; Log *K*_ow_ = 6.51) and lindane (Log *K*_ow_ = 3.72) mixture onto weathered polyethylene MNPs; (ii) evaluate the fate of the pesticide mixture coexposed with MNPs in plastic and glass exposure systems; and (iii) determine the bioavailability and cytotoxicity of the pesticide mixture coexposed with MNPs using the RTgutGC cell line. As mentioned previously, polyethylene relevance in environmental and biological samples relays on its environmental prevalence and ability to adsorb contaminants more readily than other polymers such as polystyrene.^50,51^ We selected lindane and DDE as model contaminants due to their environmental persistence, hydrophobicity, relevance as legacy pollutants in aquatic ecosystems, and bioaccumulation potential in aquatic organics.^12,52^ Moreover, these pesticides are detectable at ng/L to μg/L concentration ranges^53,54^ and represent appropriate chemicals to study coexposures of MNP with pesticide mixtures which are lacking. Our study highlights the role of environmental weathering on MNPs sorption to a pesticide mixture and their impact on pesticide and MNP's bioaccumulation and toxicity in fish intestinal cells.

## 2 Methods

### 2.1 Sample Preparation and Pesticide Chemical Confirmation

Pristine and oxidized high-density polyethylene (HDPE) MPs and a MNP mixture has been described previously for UV aging, characterization (including dynamic light scattering (DLS) and scanning electron microscopy analysis), and exposure sample preparation.^31^ Additionally, poly diversity indices (PDI) and ζ-potential were measured using a Zetasizer, Ultra (Malvern Panalytical) (Table S1). PDI data suggest that MPs and UV-aged MPs were moderately polydisperse, OX MPs and UV-aged OX MPs were monodispersed, and MNPs and UV-aged MNPs were highly polydisperse. UV weathering approaches we selected according to previous protocols.^31,55–58^ Briefly, UV aging was performed using a 340 nm UV-A lamp for 32 days following the aforementioned protocols for accelerated weathering simulating long-term environmental exposure.

All plastic and chemical sample preparations and analysis were performed in glass vials with poly­(tetrafluoroethylene) (PTFE) lined caps (Qorpak with Thermoset F217, Global Industrial). All liquid transferring was performed using glass Pasteur pipettes or graduated glass pipettes (Millipore Sigma). The particles were prepared by weighing the mass of each separate plastic type needed in a precision balance (Mettler Toledo XP2U, USA) and suspending the plastics in the exposure media, L-15/ex, which consists of similar salts content to Leibovitz’s medium (Thermo Scientific, Waltham, USA) but without amino acids and vitamins.^59^ Plastic particles and pesticide structure and formula can be found in the Supporting Information (Table S2). DDE and lindane were prepared by dissolving neat material into a master stock of 100% dimethyl sulfoxide (DMSO) and spiked to a final sample concentration of organic chemicals with 0.5% DMSO. After chemical spiking, exposure solutions were placed on an orbital shaker for 48 h (h) to allow the sorption of the pesticides. Exposure solutions were then sonicated for 20 min in an ultrasonic water bath before cell line exposure or analytical measurements to ensure sample homogeneity. Pesticide chemical concentrations for all experiments were measured using 1:1 v/v of media with hexane with liquid–liquid extraction, and the resulting hexane extract was analyzed using gas chromatography-mass spectrometry (GC–MS; 5975c; Agilent Technologies). All measured concentrations of initial exposure solution recoveries were within 10% of nominal values for both lindane (101 ± 2.5%) and DDE (104 ± 3.7%) for the sample preparations with and without particles (*n* = 9). Lindane and DDE concentrations of 2200 and 32 ng/mL, respectively, were chosen to maintain a low nonlethal dose relevant to RTgutGC cells (Figure 1) as well as maintaining solubility (7300 ng/mL for lindane and 120 ng/mL for DDE) for the exposure scenarios.

**Figure 1:**
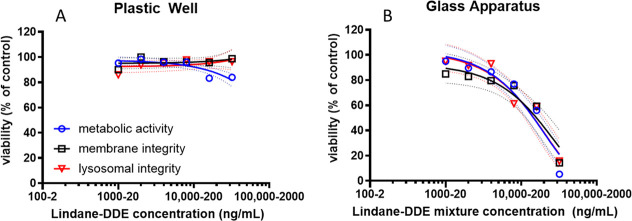
Dose–response curves of lindane and dichlorodiphenyldichloroethylene (DDE) mixture exposed to RTGutGC cells. Panel (A) represents cells seeded onto polystyrene multiwell plates and exposed for 24 h. Panel (B) represents cells seeded onto glass coverslips and exposed in glass Petri dishes for 24 h. Results from viability assays are reported as percent viability based on the fluorescent units of the L-15/ex control medium. Cytotoxicity assays of metabolic activity (alamarBlue) are indicated in blue (circles), membrane integrity (CFDA-AM) in black (squares), and lysosomal integrity (neutral Red) in red (triangle). Values reported as mean (marker) and confidence intervals (dashed lines) of at least three independent experiments (*n* = 3).

### 2.2 Characterization of PE Particles

DLS and the ζ-potential: the particle size and surface electrical charge were measured by DLS and ζ-potential analysis (ZetaPALS analyzer, Brookhaven Instruments Co., USA). The concentrations of 10 mg of MPs/L and 1 mg of UV and non-UV-weathered MNPs were measured alone along with the mixture concentration of lindane and DDE, at 2200 and 32 ng/mL, respectively. All ζ-potential determinations were performed for all particle types and treatments at a concentration of 25 mg of plastic/L (*n* = 5).

Attenuated total reflectance—Fourier transformed infrared spectroscopy: the functional chemistry of the samples was evaluated using attenuated total reflectance-Fourier transform infrared (ATR-FTIR) spectroscopy to evaluate the effects of the UV aging process and organic mixture addition on the particle types. To obtain the spectra, a Nicolet iN10-MX micro-FTIR (Thermo Fisher Scientific, USA) was used in the attenuated total reflection (ATR) mode with a cooled detector and a germanium ATR tip. Additional details can be found in the Supporting Information.

### 2.3 Adsorption of Pesticides onto MNPs

Adsorption kinetics were carried out to determine the equilibrium time of the pesticide mixture to the particles, and pesticides were spiked at 2200 and 32 ng/mL for lindane and DDE, respectively. Sample aliquots were taken and measured at 0, 6, 24, and 48 h. Samples were filtered by passing 4 mL of a 25 mg/L plastic solution suspension through a 0.22 μm cellulose filter, and the aliquot was measured for chemical concentrations. All particles were recovered and analyzed with ATR-FTIR and SEM/EDS before and after the sorption process, as described above.

### 2.4 RTgutGC Cell Culture

The immortal cell line RTgutGC was provided by Kristin Schirmer and cultured for assays as previously described.^60^ Briefly, cells were cultured in 75 cm^2^ flasks (Greiner Bio-One, Monroe, USA) at 19 °C in Leibovitz’s L-15 medium (Thermo Fisher Scientific, USA) supplemented with 5% of fetal bovine serum (FBS, Sigma-Aldrich, USA) and 1% of gentamicin (10 mg/mL, Gibco, Thermo Fisher Scientific, USA). For the preparation of exposures, cells were washed twice with Versene (Thermo Fisher Scientific, USA) and detached from the flasks using 0.25% Trypsin (Thermo Fisher Scientific, USA). Then, cells were resuspended in L-15/FBS medium, and viability and cell numbers were determined using an automatic cell counter (Countess II Automated Cell Counter; Life Technologies Corporation, USA). Only cell suspensions with a viability greater than 90% were used for experiments.

### 2.5 Cytotoxicity Assays

For all cytotoxicity assays, RTgutGC cell monolayers were seeded at a density of 80,000 cells/cm^2^. Cells were seeded into both a plastic 24-well plate (Greiner Bio-One, USA) and onto 22 mm glass collagen Type I Cellware coverslips (Corning BioCoat, USA) and were incubated for 48 h at 19 °C. For coverslips, 500 μL of cell solution was added as a droplet to the top of the coverslip and allowed to incubate for 24 h to allow for initial attachment (Figure S1). At 24 h, 2 mL of complete media was added onto the coverslips placed in glass Petri dishes and then allowed to incubate for an additional 24 h. Cells in the Petri dishes and 24-well plates were then washed twice with L-15/ex and exposed to the DDE and Lindane chemical mixture with and without PE particles in solution.

Previous studies have shown that MNPs alone were nontoxic upon exposure to RTgutGC.^31^ To determine a dose–response, pesticide mixture concentrations ranged from 2–2000 and 100–100,000 ng/mL for DDE and lindane, respectively. The concentrations of organic pesticides mixture inhibiting cell viability of 20% (or effective concentration 20%; EC20) were 150 and 7,500 ng/mL for DDE and lindane, respectively, and used for exposure with each of the MNPs with increasing plastic concentrations of 0.1, 1, 12.5, 25, 50, 100, and 200 mg/L, prepared as described in Section 2.1. RTgutGC cells exposed to L-15/FBS (i.e., complete culture medium) and L-15/ex with 0.5% DMSO (i.e., the exposure medium) served as negative controls for the viability assays. All experiments included a positive control of copper sulfate at 500 ng/mL as ion, which is known to induce an effective concentration of 50% (EC50) for RTgutGC cells.^61^ All concentrations were repeated at least in triplicate (*n* = 3). The coverslips were placed into glass Petri dishes for the exposure duration, and both plastic multiwells and glass Petri dishes were exposed at 19 °C for 24 h. After the 24 h exposure duration, cell monolayers were washed twice with L-15/ex to remove excess chemical in solution. The coverslips were transferred to a separate 24-well plate for the multiple cellular end point viability assay to be performed along with the 24-well seeded plates. The assay consisted of three fluorescent indicator dyes, alamarBlue (Invitrogen, Eugene, OR, USA), CFDA-AM (5-carboxyfluorescein diacetate acetoxymethyl ester, Invitrogen, Eugene, OR, USA), and Neutral Red (Invitrogen, Thermo Fisher Scientific, USA), which indicate cell metabolic activity, cell membrane, and lysosomal integrity, respectively. Fluorescence of alamarBlue, CFDA-AM, and Neutral Red were measured at an excitation/emission of 530/595, 485/530, and 530/645 nm, respectively, using a Cytation 5 Plate Reader (BioTek, USA). Viability results are reported as % cell viability compared to the L-15/ex with 0.5% DMSO negative control.

### 2.6 Chemical Bioaccumulation

RTgutGC cells were seeded at 80,000 cells/cm^2^ into glass Petri dishes and incubated at 19 °C for 48 h. Cells were washed twice with L-15/ex and exposed for 24 h to 25 mg/L each of the MNPs with the mixture concentration of DDE and lindane at 32 and 2200 ng/mL, respectively (e.g., which was not toxic for RTgutGC, see Figure 1). Cells were washed with L-15/ex and then lysed for 2 h at 19 °C in 1 mL of 50 mM NaOH (Sigma-Aldrich, St. Louis, MO, USA). Cell lysates were stored in Eppendorf tubes, and 0.1 mL from each sample was frozen at −20 °C and stored for protein quantification using the Lowry Assay (Thermo Fisher Scientific, Waltham, MA, USA). The remaining volume of cell lysate was mixed with hexane using a 1:1 v/v ratio for liquid–liquid extraction, and the resulting hexane extract was analyzed using gas chromatography–mass spectrometry (GC–MS; 5975c; Agilent Technologies). To consider differences in cell number in different wells, lindane and DDE accumulation data were presented as ng chemical per mg of protein determined from the same samples.

### 2.7 Cell Imaging and Plastic Uptake through Fluorescence Measurements

Cells were seeded, incubated, and exposed as previously mentioned for cytotoxicity assays into 96 well flat-bottomed cell culture plates (TPP Techno Plastic Particles, Switzerland). Pristine and UV treated NPs were fluorescently labeled following a previous method.^62^ Briefly, a working stock of 200 mg/L NPs in methanol with 5% 3-Aminopropyltrimethoxysilane (APMS; Sigman Aldrich, USA) was sonicated for 20 min and then shaken for 24 h. The 5% APMS and NP solution was filtered using a 0.1 μm polytetrafluoroethylene (PTFE) filter (Advantec, Cole Palmer, USA), rinsed in triplicate with deionized (DI) water, and then resuspended into a 1:50 solution using a working concentration of 10 μg/mL 5/6-fluorescein isothiocyanate (FITC; Thermo Scientific, USA) and methanol and sonicated for 20 min followed by shaking for 24 h. The FITC solution was then filtered off using a 0.1 μm polytetrafluoroethylene (PTFE) filter and washed in triplicate with DI water. The NPs were then resuspended in L-15/ex, sonicated for 20 min, and then diluted in L-15/ex to the final exposure concentrations of 12.5 mg NPs/L.

Cells were stained using two fluorescent dyes that stain nuclei (NucBlue, Live ReadyProbes, Thermo Fisher Scientific, USA) and lysosomes (LysoTracker Deep Red, Invitrogen, Thermo Fisher Scientific, USA) and were stained following the manufacturer’s instructions. Briefly, cells were exposed to LysoTracker Deep Red at a concentration of 70 nM and incubated for 1 h. NucBlue was added at a ratio of two drops per 1 mL and incubated for 10 min before imaging the cells. Cells were then washed twice with PBS and lysosomes, nuclei, and plastics were imaged using a plate reader (Cytation 5; BioTek) using the Cy5 (647/668), DAPI (377/447) and GFP (469/525) filters, respectively. Additional characterization (i.e., ATR-FTIR, DLS, and ζ-potential data of PE MNPs after FTIC-APMS staining) can be found in Supporting Information (Figure S2 and Tables S3 and S4).

### 2.8 Data Analysis

Statistical analysis was performed using GraphPad Prism Version 10.0 (GraphPad Software Inc., San Diego, CA). All reported data were assessed and confirmed for normality with the D’Agostino & Pearson normality test. Analysis of variance (ANOVA) followed by Dunnett’s or Tukey’s post hoc test was performed to determine the statistical significance among different experimental groups. A Student’s *t*-test was performed to evaluate significant differences when only two groups were analyzed. A minimum of three replicates were conducted for all experiments. All statistical analysis was carried out using an alpha level of 0.05 or lower and is notated within their respective figures and graphs.

## 3 Results and Discussion

### 3.1 Agglomeration, Surface Charge, and Functional Chemistry of MNPs

Differences in agglomeration for particles dispersed in exposure medium L-15/ex were measured before and after UV aging (Figure 2). Previous results show that using the same type of MNPs indicated that weathering (i.e., UV aging and chemical oxidation) can increase size through agglomeration but not that of MPs alone. Moreover, UV radiation modified the ζ-potential of oxidized MPs approximately 2-fold for each treatment.^31^ ATR-FTIR analyses indicated that UV weathering modified the functional chemistry of the MNPs (Figure 3). Peaks associated with C–O (1600–1900 cm^‑1^) appeared in Ox MPs and NPs for all conditions at approximately 1600–1900 cm^‑1^ and R–O groups signal showed for all Ox MPs (850–1000 cm^‑1^) and UV-weathered NPs (850–1300 cm^‑1^) compared to a lack of peaks in this region in the nonoxidized MNPs. Previous studies have shown that UV aging and chemical oxidation modify MNPs morphology, functional chemistry, particle size, and charge.^32,63,64^

**Figure 2:**
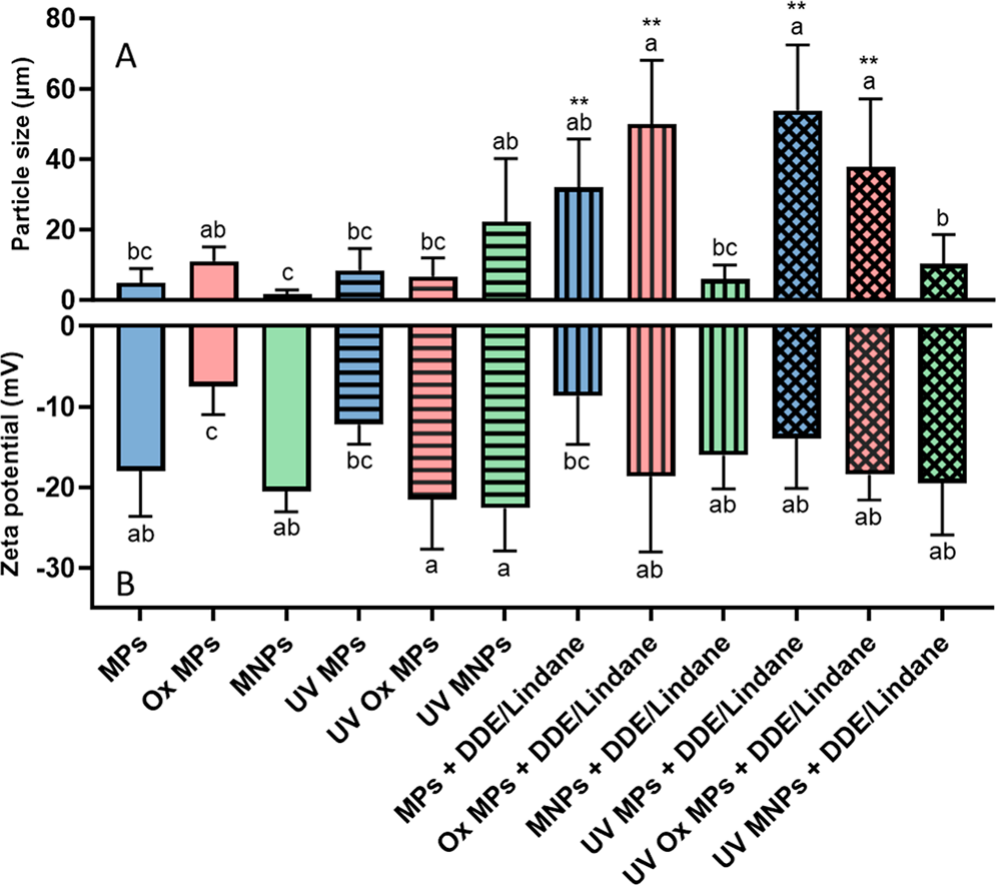
DLS (A) and ζ-potential (B) analysis of high-density polyethylene (HDPE) microplastics (MPs); oxidized MPs (Ox MPs); a mixture of micro and nanoplastics (MNPs); UV-weathered MPs; UV-weathered Ox MPs; and UV-weathered MNPs before (0 h) and after (48 h) pesticide mixture exposures (lindane and dichlorodiphenyldichloroethylene [DDE], 2200 and 32 ng/mL, respectively). Data are presented as mean ± SD. Lower case letters indicate a significant difference between particle type and exposure conditions (one-way ANOVA, Tukey’s post hoc, multiple comparison test; alpha = 0.05; *n* = 3). Asterisks indicate significant differences from particles alone in comparison to respective particles with pesticide exposures (unpaired student’s *t* tests, where *, **, and *** represent *p*-values <0.05, 0.01, and 0.001, respectively).

**Figure 3:**
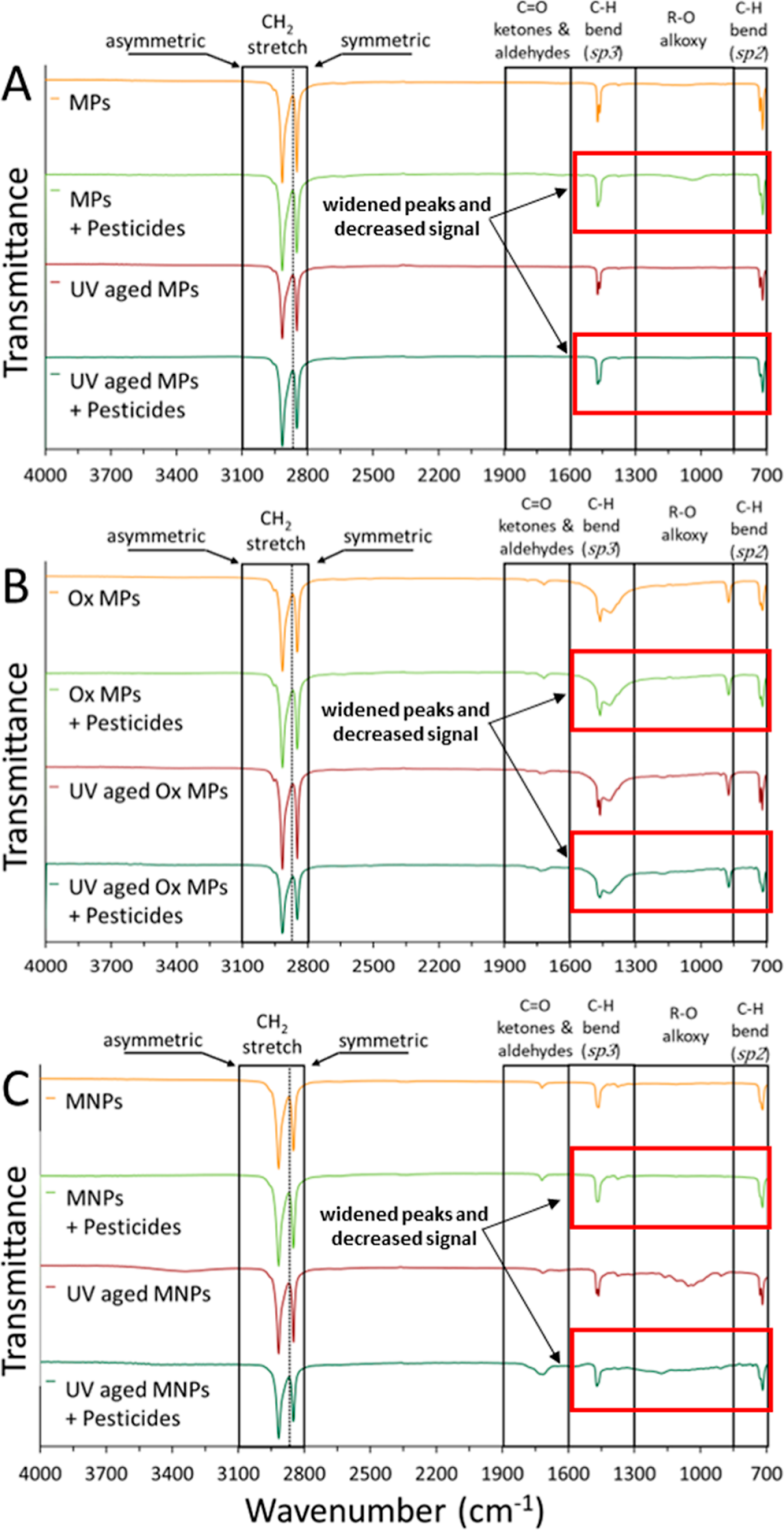
Attenuated total reflectance- Fourier transformed infrared spectroscopy (ATR -FTIR) analyses of UV and non-UV-weathered microplastics (MPs; panel A), oxidized microplastics (Ox MPs; panel B) and a mixture of micro- and nanoplastics (MNPs; panel C) before (0 h) and after (48 h) pesticide mixture adsorption (lindane and DDE, 2000 and 40 ng/mL, respectively). Arrows and red boxes indicate changes in peak width and decreasing signal between plastics with and without pesticide mixtures.

### 3.2 Adsorption of Lindane and DDE onto MNPs

Overall, the adsorption experiments indicated two main trends in the MP and MNP mixtures: (1) UV-weathering reduced the adsorption capacity of MNPs and (2) DDE adsorbed to a greater extent than lindane (Figures 4, and S2). Specifically, non-UV-weathered MPs adsorbed 35 ± 6% of the lindane supplied, whereas UV-weathered adsorbed only 9.7 ± 5% of the lindane supplied. For DDE, non-UV-weathered MPs adsorbed 69 ± 3% and UV-weathered adsorbed 63 ± 2%. These findings indicate that the UV weathering modified the sorption capacity of the PE MNP to pesticides agreeing with previous studies that assessed the sorption of lindane onto PS MPs (Cai et al., 2024).^32^

**Figure 4:**
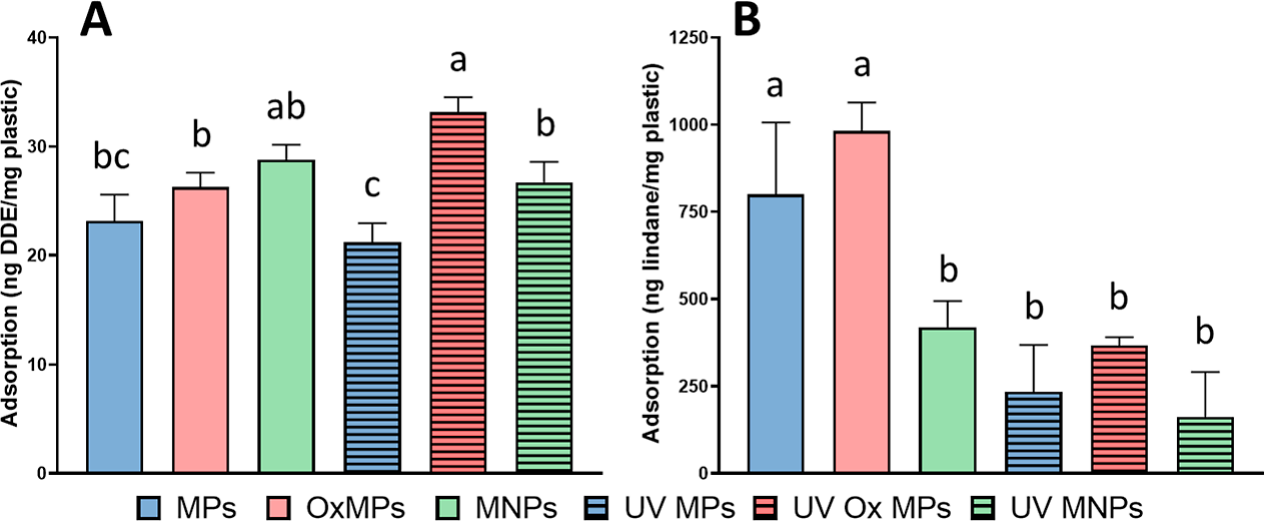
Adsorption concentrations of (A) dichlorodiphenyldichloroethylene (DDE) and (B) lindane solutions exposed to 25 mg/L various conditions of plastics up to 48 h. Conditions included DDE and lindane with microplastics (MPs); oxidized MPs (Ox MPs); a MNP mixture (MNPs); UV-aged MPs; UV-aged Ox MPs; and UV-aged MNPs. Results are reported as average ± standard deviation. Lower case letters indicate significant differences between plastic conditions and each chemical (one-way ANOVA, Tukey’s post hoc, multiple comparison test; alpha = 0.05; *n* = 3).

Desorption/sorption dynamics likely explain discrepancies in the sorption capacity. For instance, most of the treatments revealed a higher concentration of pesticides in the aqueous phase at time 48 h compared to the measurement taken at 24 h, which indicates an ongoing desorption process or a weak adsorption onto the surface of the MNPs. There were no correlations when comparing the size and charge variables across the different plastic types (Figure S4). Therefore, these parameters cannot explain our findings. A weak correlation between particle characteristics and adsorption suggests that hydrophobic partitioning may be the dominant mechanism compared to electrostatic interactions and particle size despite their impact on surface area as previously shown in Cai et al. (2024).

The two pesticides that we selected for our study display moderate and high hydrophobicities (DDE, log *K*_ow_ = 6.51; lindane, log *K*_ow_ = 3.72). Our results indicate differences in adsorption onto MNPs, demonstrating polarity, and *K*_ow_ values need to be considered when evaluating environmental partitioning and exposure of MNPs. Contrary to our results, Pinto et al. (2024)^31^ using the same MNP found that Cd adsorbed more onto UV-aged MNP in comparison to non-UV-aged MNPs which indicates that the sorption process depends on the specific affinity between MNP and the compound of interest. Further studies evaluating the breadth of potential interactions with MPs across contaminant properties including polar pesticides and antibiotics would provide greater insight into the role MPs may play in contaminant environmental fate and toxicity. Previous studies have demonstrated a rapid adsorption process of pesticides onto PE MPs^65^ and highlighted plastic ability to prolong pesticides residence times in aqueous environments.^12^ Moreover, the Log *K*_ow_ of DDE and lindane, 6.52 and 3.72, respectively, corresponds with the affinity of pesticides to MNPs in our study (Figure 4 and Table S5). Indeed, hydrophobicity and H-bonding can influence the adsorption process as previously measured in PE.^66^ Particle agglomeration increased for all plastic types excluding UV-weathered NPs increased after coexposure with pesticides which coincides with a diminished sorption capacity. Moreover, comparisons of the adsorption of each pesticide and both size and ζ-potential showed no correlation between size or ζ-potential and pesticide sorption (Figure S4), suggesting that the main mechanism of adsorption does not depend on these two parameters.

The occurrence of reduced adsorption of the terbuthylazine onto weathered PE has been previously correlated with increasing hydrophobicity and negatively charged sites caused by the formation of oxygen-containing functional groups produced from UV weathering.^67^ Therefore, our results highlight that environmental weathering, especially UV radiation, affects PE MNP affinity toward environmental pollutants depending on the intrinsic characteristics of the compound; e.g., increased affinity to metals such as Cd^31^ or decreased affinity sorption of hydrophobic chemicals such as, alachlor, methomyl^32^ and a lindane and DDE mixture (this study).

ATR-FTIR analyses indicated that the addition of the pesticide mixture decreased the signal and widened peaks (C–H sp3, 1300–1600 cm^‑1^; R–O 800–1300 cm^‑1^ and C–H sp2, 700–800 cm^‑1^) compared to the control without adsorbed pesticides (Figure 3). Sorption of pesticides onto UV-weathered MNPs decreased the signal and widening peaks in the nonreacted MNPs. R–O alkoxy peaks (900 cm–1), C=O ketones, and aldehyde groups (1700 cm–1) appeared in Ox MPs. Ketone groups were also present in the Ox MNPs. UV aging has produced similar effects in previous studies.^31^ The effect of oxidation on covalent bonds between the carboxyl and alkoxyl groups could favor potential chemical adsorption of the pesticides onto the MNPs; however, previous studies have shown that the adsorption of various pesticides did not result in additional peaks in the spectra, concluding physical adsorption may ultimately be driving partitioning onto PE particles.^65^

Previously, Cai et al. showed that the adsorption of lindane onto UV-aged and non-UV-aged polystyrene MPs followed a Freundlich model with a Freundlich constant (*K*_F_) of 0.3 and 0.52 for UV-aged and non-UV-aged, respectively.^32^ Other studies have also demonstrated that plastic can adsorb DDE; therefore, adsorption of DDE onto MNP is also expected.^68^ These previous results and our findings indicate a heterogeneous adsorption on the MNPs surface (Figures 4 and S3). Further studies should evaluate the adsorption–desorption mechanisms and sorption kinetics of contaminants such as pesticides onto various types of MNPs.

### 3.3 Cytotoxicity and Bioaccumulation

#### 3.3.1 Exposure Approach and Dose–Response of the Organic Mixture

To evaluate whether the substrate (plastic vs glass) influenced cell viability, this parameter was tested side by side. Cells were shown to grow similarly in plastic and glass systems (Figure S5). Additionally, all cellular end points, cell metabolic activity, cell membrane, and lysosomal integrity were not statistically different in cells cultured on glass or on plastic wells (Figure S6). Regardless of the efficiency of cell attachment and viability between plastic and glass systems, data is normalized to the respective control (i.e., cells exposed in absence of chemicals). Moreover, our results have indicated that the use of an all-glass exposure apparatus is necessary due to the fate of hydrophobic chemicals in plastic systems which reduce the bioavailability of such chemicals which justifies the additional costs and handling needs associated with this procedure. To determine nonlethal exposure concentrations of the pesticide mixtures, dose–response curves in RTgutGC cells were initially conducted in polystyrene culture wells. The results showed overall almost negligible cytotoxicity with only a slight decrease in viability for metabolic activity at the highest concentration tested (i.e., 30,000/300 Lindane/DDE ng/mL) (Figure 1A). However, cells were exposed using a glass exposure system (Figure S1) at the same concentration range, results indicated an acutely toxic dose dependent response for all three end points (Figure 1B). This result is due to the compounds’ hydrophobicity and partitioning out of the exposure solution into the plastic wells (Table S6) which is occurring although the chemicals were dissolved in L-15/ex supplemented with 0.5% v/v of DMSO. This effect has been previously documented for the poly aromatic hydrocarbon, fluoranthene and benzo­[a]­pyrene dissolved in the same medium (L-15/ex 0.5% DMSO) where the chemicals were found to partition to the wells upon exposure in polystyrene wells.^49,69^ Previous studies with embryo assays with organic chemical exposures of *K*_ow_ > 3 in PS multiwell plates, showed concentrations decreased rapidly but not for cell-based assays in similar conditions.^70^ The main difference being that the cell-based assays contained 10% FBS which allowed the protein and lipid rich medium to compensate for losses by the multiwell sorption, which reinforces our findings where exposures were performed in the absence of FBS and using L-15/ex exposure media. Therefore, future studies should consider the use of an all-glass approach when investigating the toxicological effects of hydrophobic chemicals.

#### 3.3.2 Cytotoxicity with MNPs and Pesticide Mixture

Based on the cytotoxicity results (alamarBlue assay–metabolic activity) obtained in the glass exposure system, the pesticide mixture EC20 (using metabolic activity) was measured at 7500 and 150 ng/mL for lindane and DDE, respectively. The EC20 was chosen to allow measurable (i.e., statistically different from controls) effects without compromising the cell’s overall viability. Therefore, to evaluate the role of MNPs on pesticides toxicity, we performed cytotoxicity assays using the EC20 of the pesticide mixture with and without MNP coexposures. Along with low environmentally relevant concentrations (0.1 and 1 mg/L), high concentrations, exceeding typical environmental levels, were used (12.5–200 mg/L; Figure 5). The rationale for the selected dose was to establish clear dose–response relationships and to identify subcytotoxic and cytotoxic thresholds of cellular and metabolic disruption. This approach is consistent with in vitro toxicological testing practices,^71,72^ particularly when environmentally relevant concentrations do not induce measurable biological effects.^73^ Additionally, recent studies have indicated excessively high concentrations of MPs in marine surface waters such as 8 g/L and up to 102–103 mg/L in estuaries where euryhaline species such as rainbow trout can be found^74^ and ecological risk analysis has determined 13.8 mg/L as the most conservative safe exposure concentration.^75^

**Figure 5:**
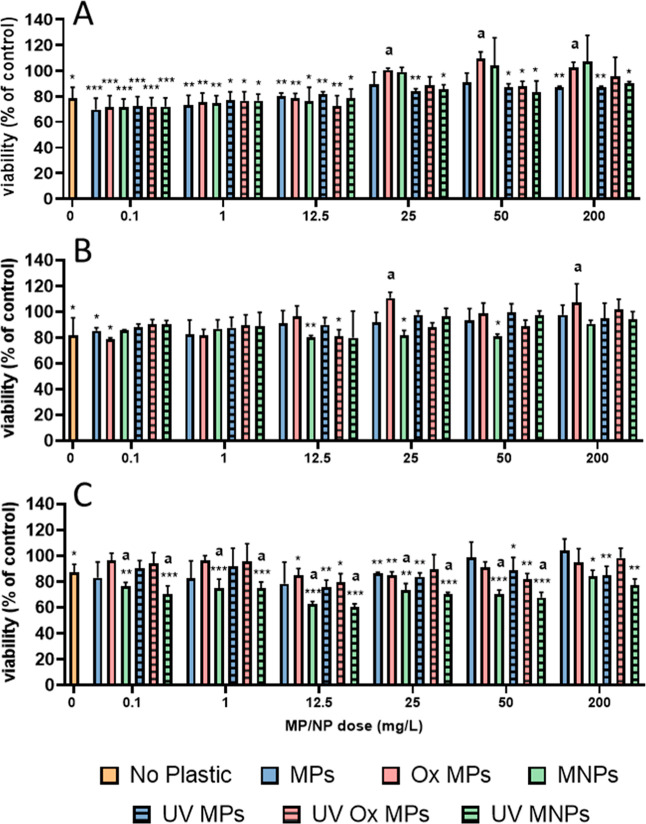
Cytotoxicity of lindane and dichlorodiphenyldichloroethylene (DDE) mixture exposures (lindane and DDE, 2000 and 40 ng/mL, respectively) to RTgutGC cells exposed in glass vessels. Viability is indicated as % control for the end points of metabolic activity (A), plasma membrane integrity (B), and lysosomal integrity (C) after 24 h exposure to a range of concentrations (12.5–200 mg/L) of microplastics (MPs); oxidized MPs (Ox MPs); a micro- and nanoplastic mixture (MNPs); UV-aged MPs; UV-aged Ox MPs; and UV-aged MNPs. Data are presented as mean ± SD. Results are reported as average ± standard deviation. Lower case letters indicate statistical difference between end point and no plastic control (one-way ANOVA, Dunnett’s multiple comparison test; alpha = 0.05, *n* = 3). Asterisks indicate statistical difference between individual end points and individual end points in L-15/ex controls (unpaired Student’s *t* tests, where *, **, and *** represent *p*-values <0.05, 0.01, and 0.001, respectively).

Positive controls reduced toxicity as expected (geometric mean of all three end points of 49.4 ± 16.9%). Increase in concentrations of MNPs reduced cytotoxicity effects (i.e., metabolic activity). This effect was particularly evident for non-UV-weathered MPs, Ox MPs, and NPs. This result corresponds well with the adsorption data where pristine plastics adsorbed more of the pesticide chemical than UV-weathered (Figure 4), thus suggesting that the reduction in cytotoxicity is driven by the MNPs sorption of the pesticides in solution. The differences in the toxicity effect could be due to variance in chemical sorption to the MPs and possibly also to the exposure system. Plasma membrane integrity (Figure 5B) indicated that excluding pristine NPs coexposure with all MNPs reduced toxicity of the pesticide mixture alone. It has previously been shown that RTgutGC cells coexposed to polystyrene nanoplastics (200 nm nanospheres) and polycyclic aromatic hydrocarbons increased DNA degradation effects induced by the 3-nitrobenzanthrone alone.^39^ Lysosomal integrity (Figure 5C) proved to be a highly sensitive end point in coexposures with MNPs especially for particles in the nano size. The neutral red assay, a well-known cellular marker of lysosomal membrane integrity^72,76,77^ indicates that both pristine and UV-aged NPs affect lysosomal integrity in fish gut cells. Previous studies have shown that silver nanoparticles affect lysosome integrity, suggesting that nanomaterials might target this cellular organelle specifically.^42^ Lysosomal integrity was affected more by pristine and UV-aged NMP than by MPs. The rationale for this result is that nanoparticles (1–1000 nm) are taken up through endocytosis and ultimately may migrate to lysosomes intracellularly.^76^ Lysosomal integrity disruption may also be occurring due to NPs presence outside or on the surface membrane of the lysosomes.^78^ While our study has shown that limited colocalization is occurring through live staining (i.e., LysoTracker and particle staining), further studies are warranted to highlight this specific mechanism (i.e., transmission electron microscope and cell fractionation studies; Figure 6).

**Figure 6:**
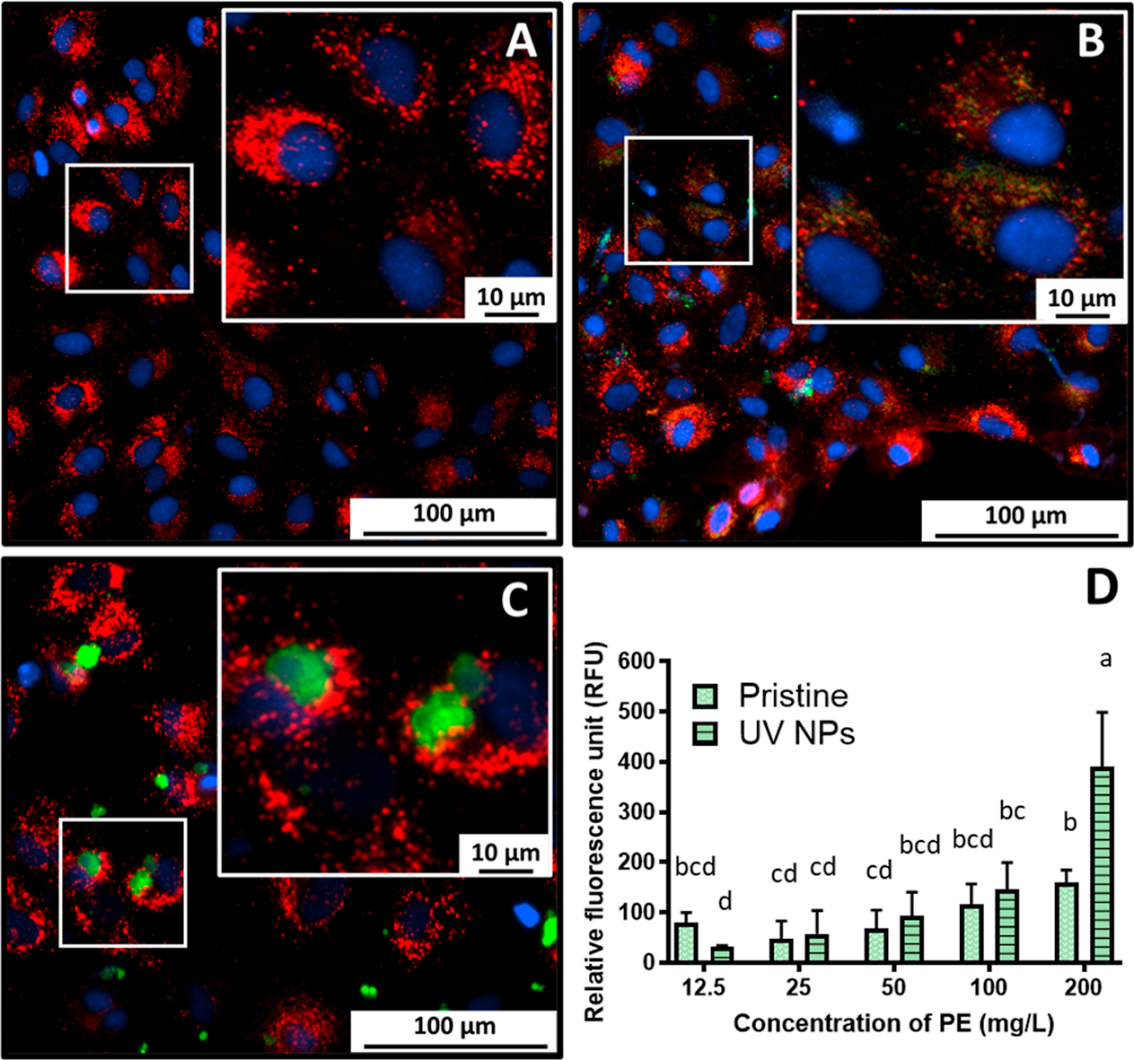
Imaging of RTgutGC cells at 20_*x*_ objective showing live staining results of nuclei in blue (nucBlue) and lysosomes in red (LysoTracker deep red) within the control (A). Green fluorescently labeled pristine (B) and UV-weathered (C) polyethylene nanoplastics were exposed at a concentration of 12.5 mg/L. Fluorescence dose–response indicating plastic accumulation in the cellular monolayer. Results are reported as average ±standard deviation. (D). Lower case letters indicate statistical difference between end point and no plastic control (one-way ANOVA, Dunnett’s multiple comparison test; alpha = 0.05, *n* = 3).

While MPs reduced the pesticide’s lysosomal toxicity in a dose-dependent manner (i.e., >50 mg/L), lysosome integrity was significantly lower in cells coexposed with MNPs and the pesticide mixture than the pesticide mixture alone; this suggests that MNPs can enter the cell and affect specifically lysosomal integrity potentiating the effect of the pesticide alone (Figure 5C). This effect is possible since particles in the nano range (<500 nm) can enter the cell by endocytosis and ultimately affect lysosomes.^79,80^ Moreover, our imaging studies using fluorescently labeled NPs support this hypothesis and show evidence of NPs internalization (Figure 6). In addition, it is evident that NPs accumulate intracellularly in a dose-dependent manner and that pristine NP accumulates in particles of smaller size compared to UV-weathered NPs (Figure 6). Previous studies have shown that lysosome integrity is impacted by NPs for a variety of human cell types including lung (BEAS-2B), intestinal (Caco-2), hepatic (HepG2), leukocytic (THP-1 and CD14+), nervous (Astrocyte 131N), and dermal fibroblast (HeLa) as well as mollusks.^80^ Ecological implications may also be of concern as lysosomal dysfunction may impair digestion and growth as well as immune responses in aquatic organisms, such as fish.^81^ Therefore, our findings suggest that nanoplastics can increase the toxicity of chemicals present in coexposure especially at the subcellular level and may have further impacts on trophic levels.

The ability for PE particles to adsorb pesticides has previously been documented.^23,34,35^ While cytotoxicity was shown to be elicited with MNP coexposures (Figure 5), DDE showed a lower uptake in the cells (Figure 7). While the sorption of pesticide onto the MNPs and reduction in acute toxicity seems to indicate that plastics may act as a protective agent to acutely toxic insults, further studies focusing on chronic exposures and effects of MNPs bioaccumulation are necessary. Moreover, it is important to note that particle size is an important factor of consideration when evaluating the effect of MNPs coexposure effect and warrant further studies on specify effect of nanoplastics. While it was beyond the scope of our study, this observation warrants future studies aimed at determining the potential chronic impacts of nanoplastics on cellular physiology and on the role, they may have on bioaccumulation and toxicity of environmental pollutants coexposed. Additionally, plastic additives such as phthalates may be of concern once accumulated within the cells.^82^ Due to the acidic environment of lysosomes, additional leaching of these chemicals may occur, resulting in the disruption of lysosomal integrity.

**Figure 7:**
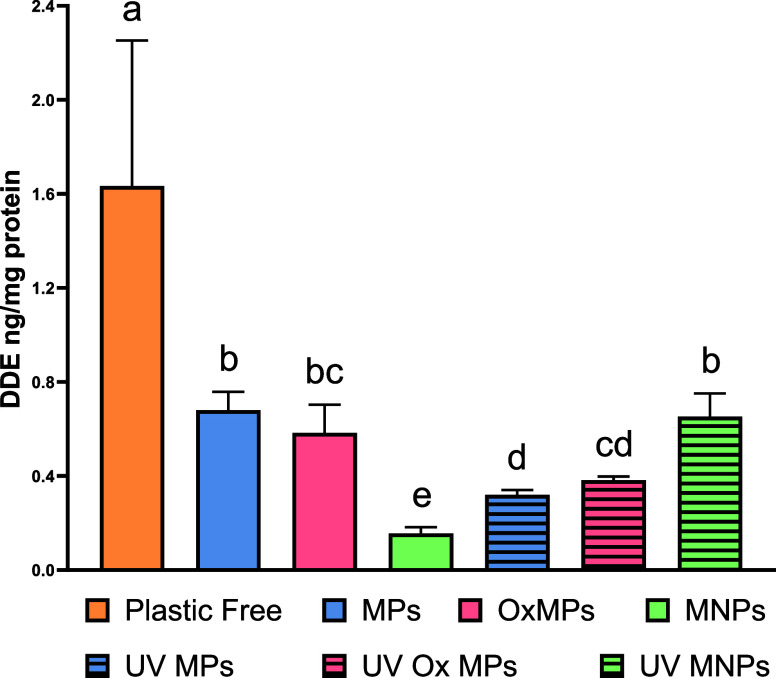
Bioaccumulation concentrations (ng/mL protein content) in RTGutGC cells exposed to 32 ng/mL dichlorodiphenyldichloroethylene (DDE) for 24 h with 25 mg/L varying conditions of plastics in solution. Conditions included (i) plastic free; (ii) microplastics (MPs); oxidized MPs (MPs Ox); a MNP mixture (MNPs); UV-weathered MPs (UV MPs); UV-weathered MPs Ox (UV MPs Ox); and UV-weathered MNPs (UV MNPs). Results are reported as average ±standard deviation. Lower case letters indicate significant difference among conditions (one-way ANOVA; Tukey’s post hoc multiple comparison test; alpha = 0.05; *n* = 3).

#### 3.3.3 Bioaccumulation with MNPs and Organic Mixtures

To evaluate the role of MNPs on pesticide bioaccumulation, RTgutGC cells were exposed to the EC20 of the pesticide mixture with and without MNPs added at 25 mg/L. The results indicated that the lindane cellular concentration was below detection. The lindane concentration was detectable and remained stable in solution and in the cell washing solutions, but it was not measured in the cells (Figure S7). Conversely, DDE cellular bioaccumulation was detectable and measured in cells exposed to the pesticide mix in the presence and absence of MNPs (Figure 7). The difference in the bioaccumulation measurement between these two pesticides could be explained by the chemical hydrophobicity and polarity of lindane and DDE where lindane is remaining in solution while DDE accumulates more readily in cells, reinforcing the mechanism that log *K*_ow_ is driving partitioning into the cells (Figure S7). The detection limits of the analytical instrument (3 ng/mL) for lindane should also be considered. Furthermore, metabolic biotransformation could not be excluded but studies looking at lindane metabolism in fish tissues are lacking. Moreover, while biotransformation may be occurring, metabolites would require identification to better determine the uptake of pollutants such as lindane as seen in previous studies using cell lines such as RTgutGC.^83^ The exposure in the absence of MNPs indicated the highest concentration of intracellular DDE. This can be expected by the partitioning of DDE to the MNPs in solution. Due to its hydrophobicity, DDE is mostly transported intracellularly via passive diffusion, and while the inhibition of transport mechanism might have a role,^84^ these data are better explained by MNP adsorption (Figure 4). For all coexposure scenarios with MNPs, a decrease in bioaccumulation of DDE was observed, suggesting that MNPs adsorb hydrophobic compounds, reducing the uptake into the cells. Interestingly, coexposures of pristine MNP mixtures, which showed the highest adsorption to DDE (86% of total; Figures 4 and S3), also showed the lowest DDE bioaccumulation.

Previous studies have also shown that coexposure of an herbicide with PE MPs reduced the toxicity of the herbicide in tilapia.^85^ Another study demonstrated that chlorpyrifos (an insecticide) coexposed with PE MPs caused a decrease in toxicity in unicellular microalgae^86^ and the presence of PVC MPs in exposure medium reduced toxicity of chlorpyrifos and triclosan an (antibacterial) in atremia salina^87^ as well as the bioaccumulation and toxicity of difenoconazole (a fungicide) to zebrafish (*Danio rerio*).^88^ Overall, our study has shown that the use of in vitro approaches is useful to measure bioaccumulation and toxicity of MNPs in complex coexposure scenarios. However, further studies should build upon our results to evaluate the role of physiologically relevant exposure such as more complex environmental matrices (e.g., presence of gastrointestinal fluids and food) to ultimately compare the effects with in vivo studies, thus strengthening the translational aspect of this work.

## Supplementary Material



## Notes

Additional materials and methods for characterization of MNPs, results for exposure chemical recovery and mass balance, and glass to plastic viability comparisons (PDF)
