# Supplementary material for: Coexposure of Micro and Nano-Plastics with Pesticides: Cytotoxicity and Bioaccumulation Effects on a Fish Intestinal Cell Line
Source: Environ Sci Technol. 2025 Dec 29;60(1):299–310. doi: 10.1021/acs.est.5c14140 (PMC12810225; doi:10.1021/acs.est.5c14140)
Supplement: Supplementary file 1 [file es5c14140_si_001.pdf]

# COEXPOSURE OF MICRO AND NANO-PLASTICS WITH PESTICIDES: CYTOTOXICITY AND BIOACCUMULATION EFFECTS ON A FISH INTESTINAL CELL LINE

**Justin Scott[1,2*], Estefanía Pereira Pinto[2,3], Kyle Forsythe[1], Kendra Hess[1], Jason Belden[2], Jorge Gonzalez-Estrella[1] & Matteo Minghetti[2]**

1: School of Civil and Environmental Engineering. Oklahoma State University, Stillwater, Oklahoma, 74078, United States of America
2: Department of Biology, Oklahoma State University, Stillwater, Oklahoma 74078, United States of America
3: Centro de Investigación Mariña, Departamento de Ecoloxía e Bioloxía Animal, Laboratorio de Ecoloxía Costeira (ECOCOST), Universidade de Vigo, Vigo 36310, Spain.

* Corresponding author
Email: justin.scott@okstate.edu

# Content:

## Supplementary Materials and Methods

Plastic Particles and Weathering Process…………………………………………………………………………………………3

Attenuated Total Reflectance - Fourier Transformed Infrared Spectroscopy……………………………………………3

**Results**

**Table S1.** Plastic particle polydiversity index……………………………………………………………………………………4

**Table S2.** Plastic particle and toxicant structure and chemical formula ……………………………………………4

**Table S3.** DLS of micro- and nanoplastics (NMPs) after FITC-APMS staining…………………………………………4

**Table S4:** Zeta potential of micro- and nanoplastics (NMPs) after FITC-APMS staining……………………………5

**Table S5**: Initial and final concentrations upon plastic adsorption……………………………………………………5

**Table S6**: Chemical recovery in cytotoxicity assays exposure medium………………………………………………6

**Figure S1:** Image of RTgutGC cell seeded on glass coverslips using a droplet approach for cytotoxicity assays. For testing the cytotoxicity assay the glass slides are transferred to a 24 well plate and cell viability is measured using the multiple endpoint assay……………………………………………………………………6

**Figure S2.** Attenuated Total Reflectance- Fourier Transformed Infrared Spectroscopy (ATR - FTIR) analyses of UV and non-UV aged micro- and nanoplastics (MNPs) after FITC-APMS staining………….7

**Figure S3:** Adsorption concentrations of lindane and dichlorodiphenyldichloroethylene (DDE) solutions exposed to 25 mg/L of various conditions of plastics for up to 48 h. Conditions included lindane (A, B) and DDE (C, D) with microplastics (MPs); oxidized MPs (Ox MPs); a mixture of micro- and nanoplastics (MNPs); UV aged MPs; UV aged Ox MPs; and, UV aged MNPs. Asterisks indicate significant differences at various time points compared to initial exposure (one-way ANOVA, Dunnett's post hoc, multiple comparison test; alpha = 0.05; n =3, where *, **, and *** represent p-values < 0.05, 0.01, and 0.001, respectively)…………………………………………………………………………………………………………8

**Figure S4:** Results comparing DDE adsorption with zeta potential (A); DDE and particle size (C); lindane adsorption with zeta potential (B); and, lindane and particle size (D). Conditions included microplastics (MPs); oxidized MPs (Ox MPs); nanoplastics (NPs); UV aged MPs; UV aged Ox MPs; and, UV aged NPs….9

**Figure S5.** RTgutGC cells seeded on plastic flat bottom wells (A) and glass cover slides (B) at 48-hour incubation on complete media……………………………………………………………………………………………10

**Figure S6:** Multiple endpoint analysis comparison between RTgutGC cells seeded on flat bottom plastic wells and glass slides at 48-h in complete culture medium. Fluorescence units indicated no significant difference between respective endpoint between plastic and glass conditions (unpaired student's t tests, alpha = 0.05)……10

**Figure S7**: Pesticide recovery of pre and post exposure solution of RTgutGC bioaccumulation studies with and without polyethylene nano- and microplastics……11

# SUPPLEMENTARY MATERIALS AND METHODS

## Plastic Particles and Weathering Process

Pristine and oxidized MPs, and the NPs were purchased from Micropowders Inc. (www.micropowders.com). We used micronized high-density polyethylene (HDPE) powder (MPP 635-XF) as the pristine MPs and constitutes a mixture of different shapes and sizes (nominal size 2-10 µm) of polyethylene particles, melting points of 123-125 °C and a density of 0.97 g/cc (at 25 °C). The oxidized microplastic (Ox MP) was a micronized powder (Aquatex 325) with a high molecular weight, nominal sizes ranging between 10–15 µm, melting points of 135–140 °C, and a density of 0.99 g/cc (at 25 °C). For MNP experiments, we acquired polyethylene micro- and nanospheres (PENS-0.95; www.cospheric.com). The nominal size range of MNPs provided by the supplier is 200-9900 nm and density at 0.95-0.98 g/cc. All three different particles were irradiated in glass Petri dishes without a lid and placed under UV light (302 nm) at 30 cm for 42 days (d) in a Bio-Rad Gel Doc XR+ Gel Documentation System (Bio-Rad, Hercules, USA) to mimic the weathering process occurred in the environment, following previous studies[29,44,45]. The particles were manually shaken daily to facilitate a homogenous UV aging process for experimental analysis and exposures.

## Attenuated Total Reflectance - Fourier Transformed Infrared Spectroscopy

Each measurement was taken using a 51 s detection time with 256 scans, a spectral range of 4000-675 cm-1 and a resolution of 8 cm-1 and collected in triplicate. The detection limit of the instrument is 20 µm. When particles were smaller than 20 µm, MPs/NPs were grouped

into a pile to be equal to or greater than 20 µm, ensuring all IR energy was directed at the MPs/NPs sample rather than background.

# RESULTS

## Figures and Graphs

**Table S1: Plastic particle polydiversity index.**

| PE Plastic Type | Polydiversity Index |
|---|---|
| MP | 0.7 |
| Ox MP | 1 |
| MNP | 0.0448 |
| UV aged MP | 0.4325 |
| UV aged Ox MP | 1 |
| UV aged NMP | 0.2505 |

**Table S2**: Plastic particle and toxicant structure and chemical formula.

| Name | Molecular structure | Chemical formula |
|---|---|---|
| Polyethelene | 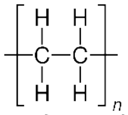 | $(C_3H_6)_n$ |
| Lindane | 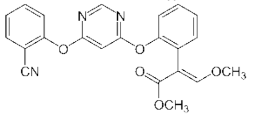 | $C_6H_6Cl_6$ |
| Dichlorodiphenyl dichloroethylene (DDE) | 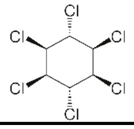 | $C_{14}H_8Cl_4$ |

**Table S3: DLS of micro- and nanoplastics (NMPs) after FITC-APMS staining.**

| Size (nm) | | |
|---|---|---|
| Trial | Pristine | UV |
| 1 | 4396 | 6164 |
| 2 | 4206 | 5532 |
| 3 | 5535 | 5206 |
| Average | 4712 | 5634 |
| StDev | 718.8 | 487.1 |

**Table S4: Zeta potential of micro- and nanoplastics (NMPs) after FITC-APMS staining.**

| Zeta Potential (mV) | | |
|---|---|---|
| Trial | Pristine | UV |
| 1 | -21.18 | -47.53 |
| 2 | -25.82 | -42.7 |
| 3 | -22.3 | -43.85 |
| Average | -23.10 | -44.69 |
| StDev | 2.421 | 2.523 |

**Table S5**: Initial and final concentrations upon plastic adsorption.

| | DDE concentration (ng/mL) AVE ± SD | | | |
|---|---|---|---|---|
| Plastic type | Time (hours) | | | |
| | 0 | 6 | 24 | 48 |
| MP | 33.1 ± 1.4 | 7.3 ± 0.2 | 2.3 ± 0.1 | 10 ± 1 |
| Ox MP | | 3.7 ± 2 | 4.1 ± 0.3 | 6.9 ± 0.5 |
| MNP | | 2.3 ± 1.8 | 2.9 ± 0.2 | 2.9 ± 4.4 |
| UV MP | | 9.9 ± 4.3 | 6.9 ± 0.3 | 12 ± 0.6 |
| UV Ox MP | | 23.6 ± 1.8 | 13.1 ± 0.8 | 11.8 ± 0.8 |
| UV MNP | | 4.2 ± 0.1 | 6.1 ± 0.9 | 6.4 ± 0.6 |

| Plastic type | Lindane concentration (ng/mL) AVE ± SD | | | |
| --- | --- | --- | --- | --- |
| | Time (hours) | | | |
| | 0 | 6 | 24 | 48 |
| MP | 2221 ± 64 | 1720 ± 11 | 1430 ± 183 | 1421 ± 143 |
| Ox MP | | 1514 ± 117 | 885 ± 68 | 1239 ± 22 |
| MNP | | 1978 ± 96 | 1334 ± 72 | 1803 ± 29 |
| UV MP | | 1944 ± 81 | 1824 ± 35 | 1987 ± 104 |
| UV Ox MP | | 1995 ± 130 | 1829 ± 114 | 1854 ± 63 |
| UV MNP | | 1820 ± 97 | 1479 ± 144 | 2058 ± 82 |

**Table S6**: Chemical recovery in cytotoxicity assays exposure medium.

| Chemical | Exposure Vessel Type | Chemical Recovery 24-h (Mean ± SD) |
| --- | --- | --- |
| Lindane | 24 well polystyrene | 32 ± 7 |
| Lindane | Glass drop technique | 83 ± 8 |
| DDE | 24 well polystyrene | 10 ± 4 |
| DDE | Glass drop technique | 72 ± 6 |

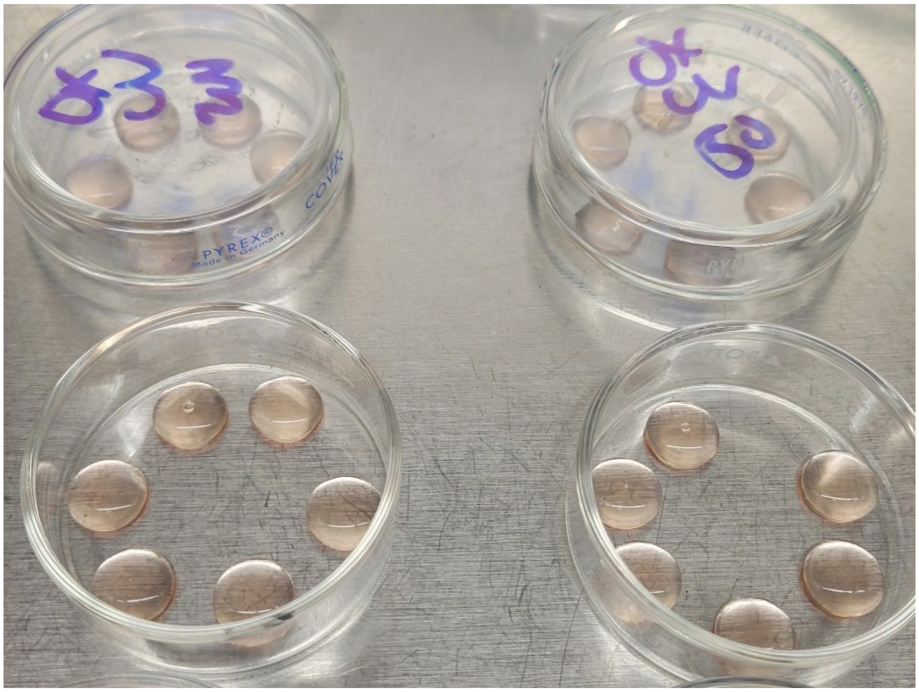


**Figure S1:** Image of RTgutGC cell seeded on glass coverslips using a droplet approach for cytotoxicity assays. For testing the cytotoxicity assay the glass slides are transferred to a 24 well plate and cell viability is measured using the multiple endpoint assay.

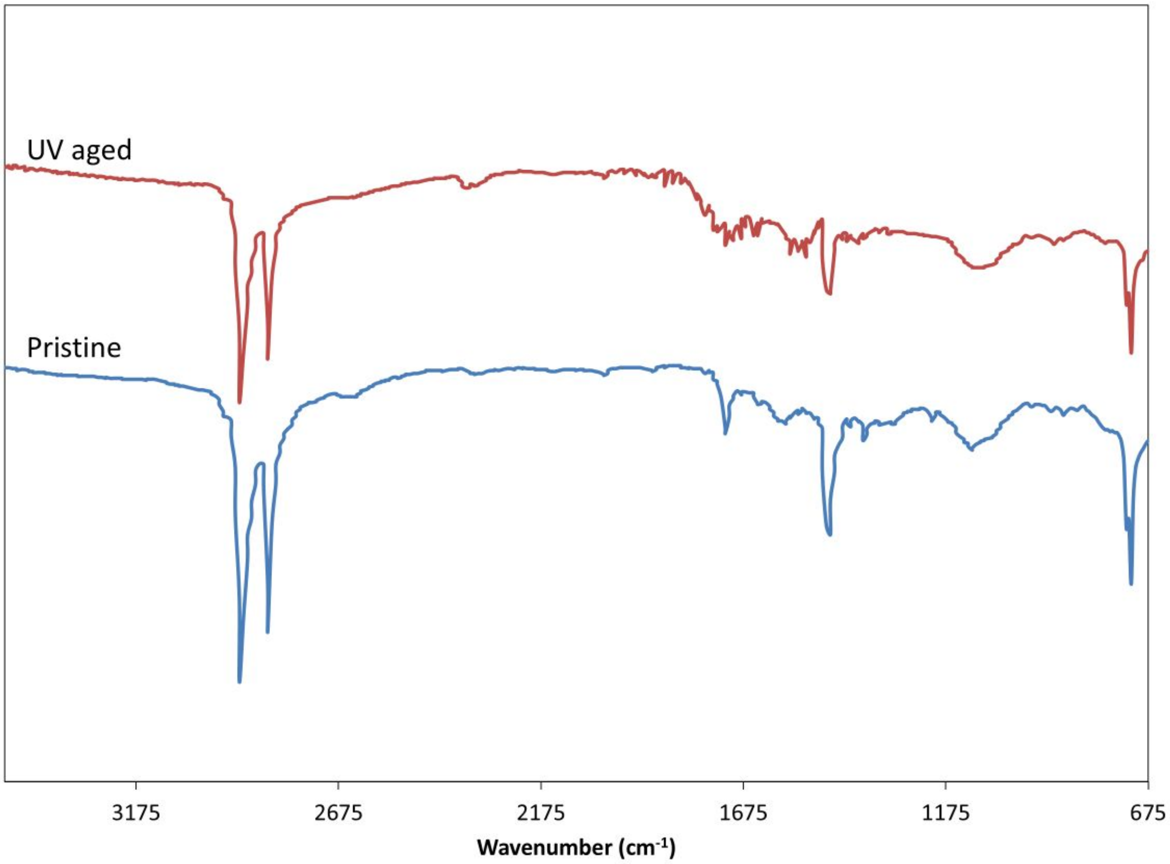


**Figure S2.** Attenuated Total Reflectance- Fourier Transformed Infrared Spectroscopy (ATR - FTIR) analyses of UV and non-UV aged micro- and nanoplastics (MNPs) after FITC-APMS staining.

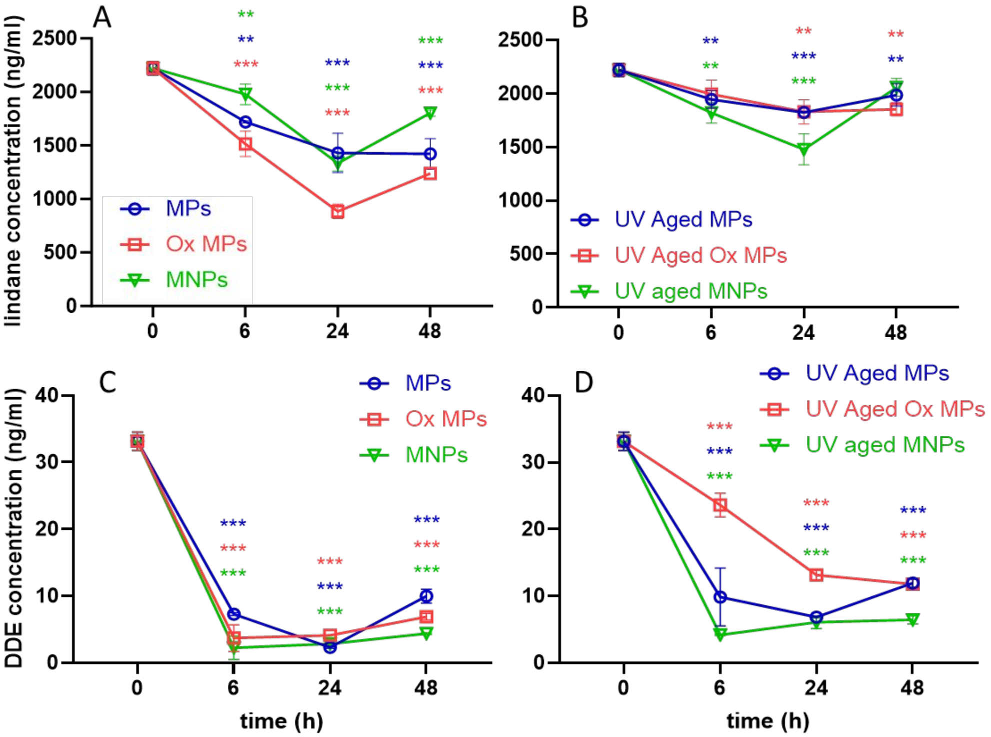


**Figure S3:** Adsorption concentrations of lindane and dichlorodiphenyldichloroethylene (DDE) solutions exposed to 25 mg/L of various conditions of plastics for up to 48 h. Conditions included lindane (A, B) and DDE (C, D) with microplastics (MPs); oxidized MPs (Ox MPs); a mixture of micro- and nanoplastics (MNPs); UV aged MPs; UV aged Ox MPs; and, UV aged MNPs. Asterisks indicate significant differences at various time points compared to initial exposure (one-way ANOVA, Dunnett's post hoc, multiple comparison test; alpha = 0.05; n =3, where *, **, and *** represent p-values < 0.05, 0.01, and 0.001, respectively).

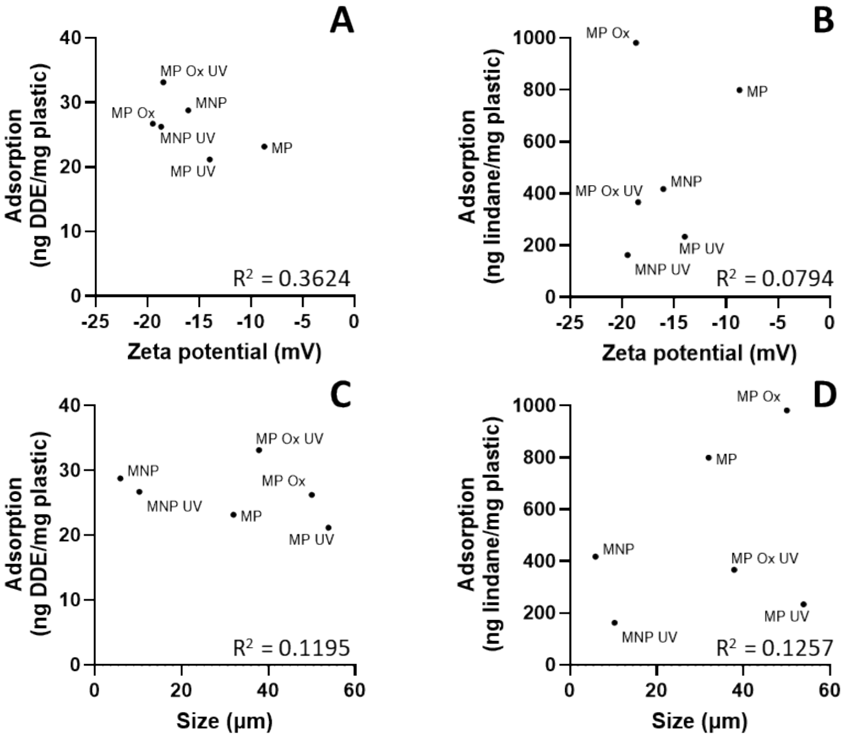


**Figure S4:** Results comparing DDE adsorption with zeta potential (A); DDE and particle size (C); lindane adsorption with zeta potential (B); and, lindane and particle size (D). Conditions included microplastics (MPs); oxidized MPs (Ox MPs); nanoplastics (NPs); UV aged MPs; UV aged Ox MPs; and, UV aged NPs.

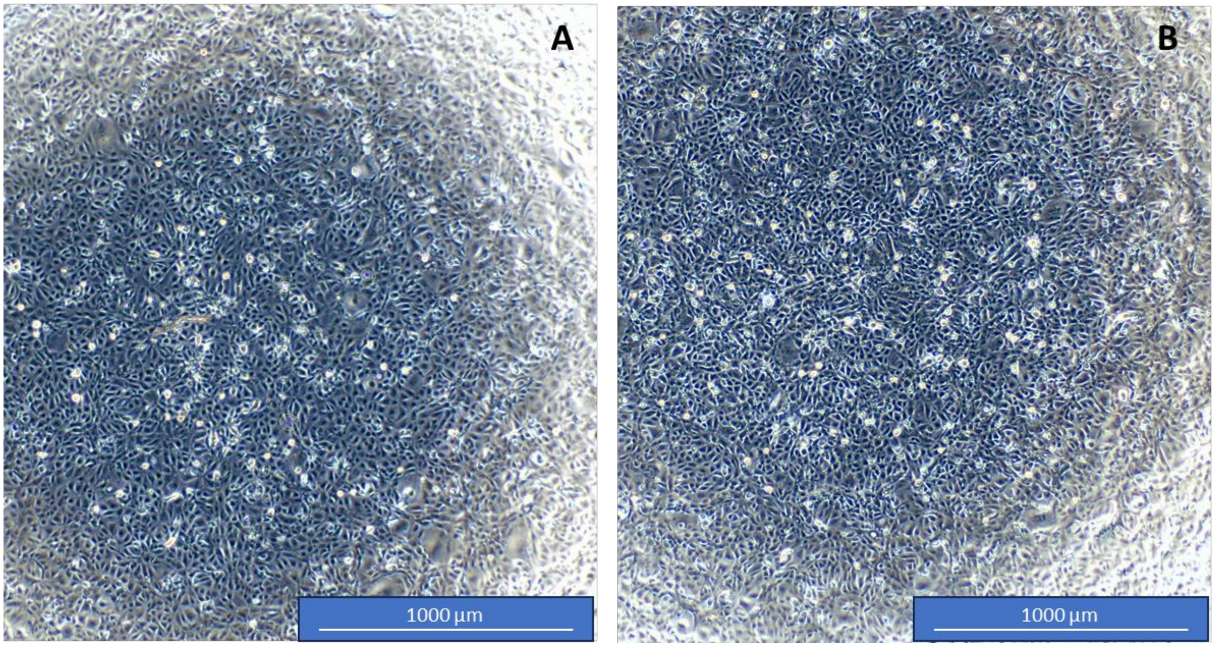


**Figure S5.** RTgutGC cells seeded on plastic flat bottom wells (A) and glass cover slides (B) at 48-hour incubation on complete media.

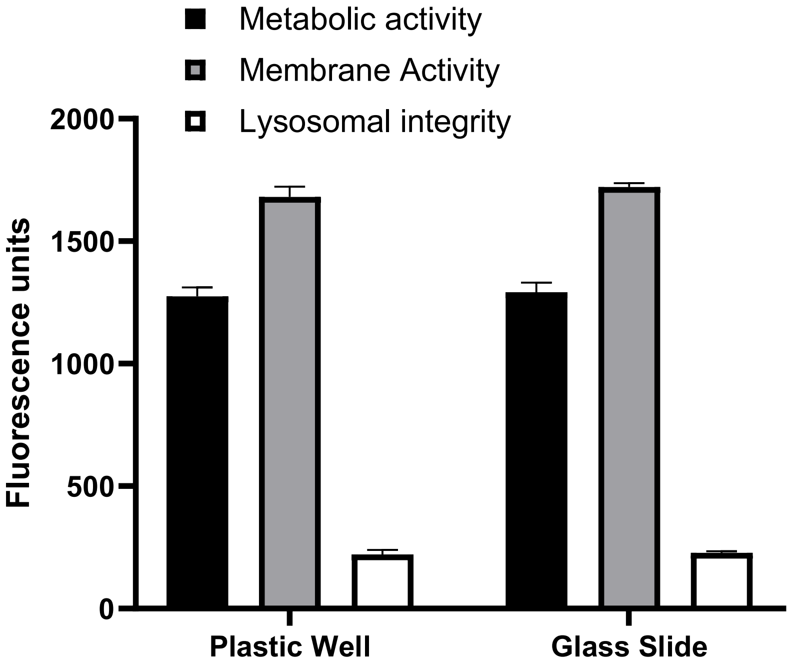


**Figure S6:** Multiple endpoint analysis comparison between RTgutGC cells seeded on flat bottom plastic wells and glass slides at 48-h in complete culture medium. Fluorescence units indicated no significant difference between respective endpoint between plastic and glass conditions (unpaired student's t tests, alpha = 0.05).

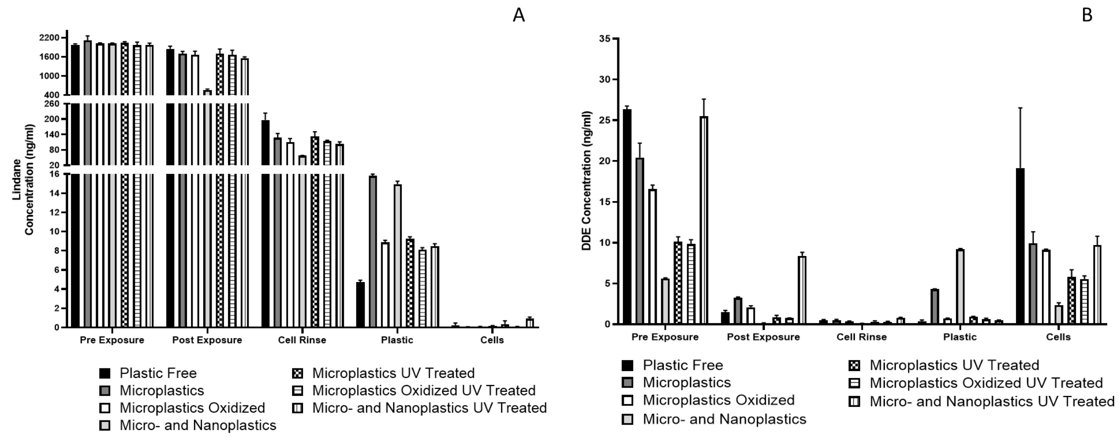


**Figure S7**: Pesticide recovery of pre and post exposure solution of RTgutGC bioaccumulation studies with and without polyethylene nano- and microplastics.
